# ROS-responsive nanocarriers for the delivery of atorvastatin in ischemic MCAO rats promoting neuroprotection

**DOI:** 10.1016/j.mtbio.2026.103296

**Published:** 2026-05-28

**Authors:** Raveena Nagareddy, Aravindkumar Sundaram, Ja-hae Kim, Ji hye Kim, Shyam Vasvani, Seho Kweon, In-Kyu Park, Yong Yeon Jeong, Kang-Ho Choi

**Affiliations:** a Department of Biomedical Sciences, Chonnam National University Medical School, 322 Seoyang-ro, Hwasun-eup, Hwasun-gun, Jeollanam-do, 58128, South Korea; b Department of Nuclear Medicine, Chonnam National University Medical School, Chonnam National University Hospital, Gwangju, 61469, South Korea; c Department of Neurology, Chonnam National University Medical School, Chonnam National University Hospital, Gwangju, 61469, South Korea; d Department of Radiology, Chonnam National University Medical School, Chonnam National University Hwasun Hospital, 322 Seoyang-ro, Hwasun-eup, Hwasun-gun, Jeollanam-do, 58128, South Korea; e DR Cure Inc, Hwasun-eup, Hwasun-gun, Jeollanam-do, 58128, South Korea; f College of Pharmacy, Chonnam National University, Gwangju, 61186, South Korea

**Keywords:** Nanocarrier, Blood-brain barrier, Hydrogen peroxide, Thioketal, Lipid peroxidation, Neuroprotection

## Abstract

Ischemic stroke remains a major global public health burden, ranking among the leading causes of long-term neurological disability and mortality. Despite advances in acute care, drug delivery to injured brain tissue is hindered by the poor permeability of the blood-brain barrier (BBB), a challenge exacerbated by excess reactive oxygen species (ROS), particularly hydrogen peroxide (H_2_O_2_), produced during ischemia-reperfusion. This oxidative surge also offers an opportunity to design ROS-responsive, site-specific therapeutic delivery systems. Therefore, this study aims to develop PEGylated thioketal-based nanocarriers (PTC) loaded with atorvastatin, a statin with both neuroprotective and anti-inflammatory properties. The thioketal linker in PTC undergoes selective cleavage by H_2_O_2_, enabling the nanocarrier to function as both a smart drug delivery vehicle and an ROS scavenger. *In vitro* experiments using oxygen-glucose deprivation-treated BV-2 microglial cells demonstrated that PTC-statin significantly lowered intracellular ROS, inhibited lipid peroxidation, and reduced inflammatory cytokine release. *In vivo*, intravenous PTC-statin administration in a rat middle cerebral artery occlusion model significantly reduced infarct size, improved motor coordination (*p* = 0.032), and enhanced neurological outcomes (*p* = 0.002), with MRI confirming infarct reduction as early as day 5 (*p* = 0.02). Molecular and histological analyses further revealed restored BBB integrity, reduced neuronal apoptosis, and promoted neurogenesis. Overall, these findings highlight PTC-statin as a precision nanomedicine that enables site-specific, ROS-triggered drug release and mitigates oxidative damage, offering a targeted therapeutic strategy for ischemic stroke recovery.

## Highlights

• New drug carriers improve recovery after a brain stroke.
• The nanocarrier system reduces brain damage and swelling.
• Treatment strengthens blood vessels in the brain.
• Drug carriers lower brain inflammation and stress.
• Promising option for future stroke therapies.

## Graphical abstract

**Figure:**
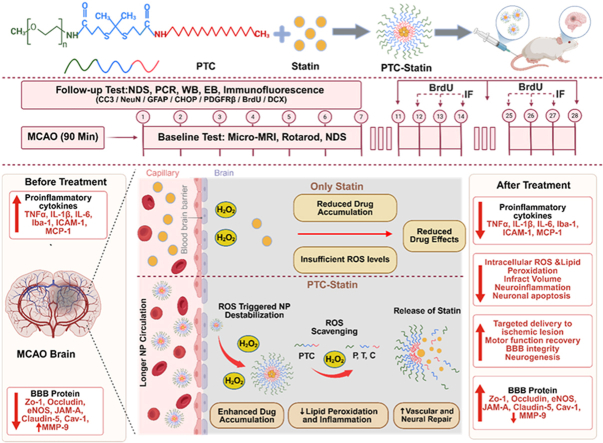

## 1 Introduction

Ischemic stroke accounts for about 85% of all strokes worldwide and is a leading cause of death and long-term neurological disability [1,2]. It results from sudden disruption of cerebral blood flow due to thrombotic or embolic occlusion, initiating a cascade of harmful processes including energy failure, excitotoxicity, calcium overload, blood-brain barrier (BBB) breakdown, inflammation, and oxidative stress [3,4]. Despite advances in acute stroke care, treatment options remain limited, particularly for patients who present outside the narrow window for early intervention. Existing treatments primarily focus on vessel recanalization but do not prevent or repair the extensive secondary damage following ischemia. This secondary injury, largely driven by oxidative stress and inflammation, highlights the urgent need for neuroprotective strategies that mitigate damage beyond the acute phase and promote long-term neurovascular recovery. Among these, interventions that target the pathological microenvironment, particularly the sustained surge of reactive oxygen species (ROS) into the subacute stage, have gained significant attention for their ability to limit neuronal loss and promote functional recovery.

A major challenge in neuroprotection is the limited delivery of therapeutic agents to the brain because of the BBB, which often remains intact or only partially disrupted after ischemic injury [5,6]. Although some strategies transiently open the BBB or exploit endogenous transport pathways, they often cause uncontrolled leakage or require extensive modification of therapeutic agents [7,8]. Nanomedicine-based approaches offer a promising alternative, particularly for ischemic stroke, where the pathological microenvironment, including elevated ROS levels, can be leveraged for targeted and controlled drug release to cross BBB [7,9].

Among the ROS species generated during ischemia, hydrogen peroxide (H_2_O_2_) is particularly abundant and drives a cascade of secondary injury, including lipid peroxidation, protein oxidation, mitochondrial dysfunction, DNA fragmentation, tight junction degradation, and activation of pro-apoptotic and pro-inflammatory signaling pathways [10,11]. Collectively, these processes exacerbate neuroinflammation, compromise BBB integrity, and accelerate neuronal death. Given this central role of ROS in both neuronal injury and BBB disruption, ROS-responsive nanocarriers that actively scavenge oxidative species while delivering neuroprotective cargo offer a particularly compelling therapeutic approach for site-specific drug delivery and concurrent oxidative stress control. Thioketal-based linkers, selectively cleaved by H_2_O_2_, allow nanoparticles to degrade specifically in ROS-rich environments [12,13]. However, most ROS-responsive nanocarriers reported to date function primarily as passive delivery platforms that exploit the oxidative microenvironment for triggered cargo release without directly attenuating the ROS burden itself [[14], [15], [16]]. Similarly, previously reported statin-loaded nanosystems, including lipid nanoparticles, have generally relied on passive enhanced permeability and retention (EPR)-based accumulation, lacking integrated ROS-responsiveness or active antioxidant functionality [17,18].

**Fig. 1:**
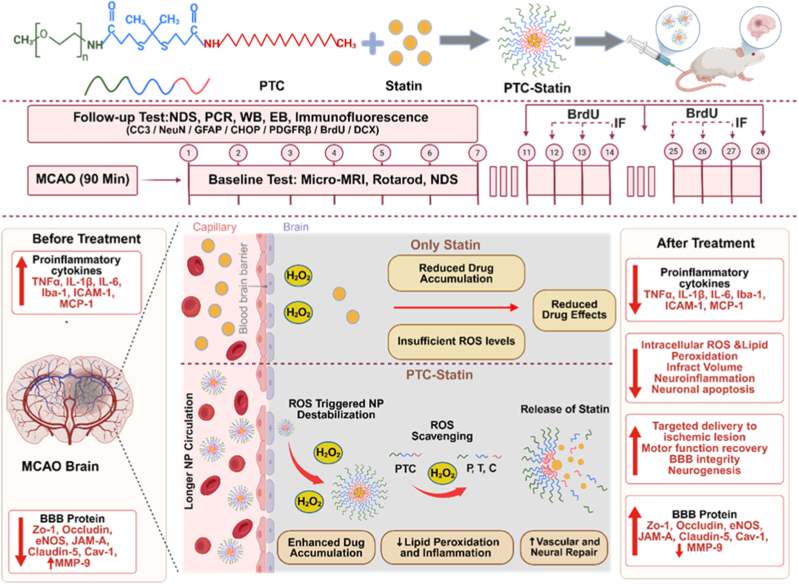
**Schematic of the design, ROS responsiveness, and therapeutic evaluation of PEGylated thioketal-based micelles (PTC-statin) for ischemic stroke treatment.** ROS, reactive oxygen species; PTC, polyethylene glycol-thioketal-C18; NP, nanoparticle; WB, Western blotting; PCR, polymerase chain reaction; NDS, neurological deficit scores; EB, Evans blue.

In this study, we developed PEGylated thioketal micelles (PTC) as dual-functional nanocarriers for targeted delivery of atorvastatin calcium, a clinically approved statin with anti-inflammatory, endothelial-stabilizing, and neuroprotective properties [19,20]. PTC micelles remain stable under physiological conditions but disassemble in response to elevated H_2_O_2_ levels in ischemic brain regions, thereby facilitating localized drug release. In contrast to conventional ROS-responsive nanocarriers designed solely for passive cargo release, the thioketal moiety in the PTC platform functions as a stoichiometric ROS scavenger during degradation. This dual mechanism, ROS-triggered release and ROS neutralization, addresses two key challenges in ischemic stroke therapy, namely poor drug accumulation in the brain and persistent oxidative damage. Therefore, this study sought to develop ROS-responsive PTC nanocarriers encapsulating atorvastatin calcium to achieve site-specific delivery and improve therapeutic outcomes in cerebral ischemia. Fig. 1 schematically illustrates this approach, including PTC-statin micelle formulation, BBB interaction, and therapeutic effects.

## 2 Materials and methods

### 2.1 Chemicals and reagents

Stearyl amine (C18), 1-ethyl-3-(3-dimethylaminopropyl)carbodiimide hydrochloride (EDC), N-hydroxysuccinimide (NHS), 3-mercaptopropionic acid, triethylamine, dimethyl sulfoxide (DMSO), and N,N-dimethylformamide (DMF) were obtained from Sigma-Aldrich (USA). Methoxy-poly(ethylene glycol)-amine (mPEG-NH_2_, 2000 Da) was sourced from SunBio Inc. (South Korea).

### 2.2 Preparation and characterization of PTC nanocarriers

The PTC nanocarrier was synthesized with slight modifications to a reported protocol [[14], [15], [16]].

#### 2.2.1 Synthesis of Thioketal (TK)

The thioketal (TK) intermediate was synthesized via acid-catalyzed condensation of acetone and 3-mercaptopropionic acid. Briefly, acetone (19 mL) was placed in a reaction flask under a nitrogen atmosphere and equilibrated at 25 °C for 10 min. Subsequently, 3-mercaptopropionic acid (31.8 mL) was added, and the mixture was stirred for 30 min. Trifluoroacetic acid (TFA, 400 μL) was then added dropwise as a catalyst, and the reaction was allowed to proceed for 5 h under continuous stirring at room temperature.

After completion, the reaction mixture was transferred into 50 mL centrifuge tubes and cooled at 4 °C for 1 h. An equal volume of deionized water was added, vortexed thoroughly, and the mixture was stored at 4 °C overnight to facilitate phase separation and precipitation. The product was collected by centrifugation (10 min), followed by repeated washing with hexane and cold deionized water to remove residual impurities.

#### 2.2.2 Synthesis of PEG-TK (Intermediate conjugate)

For PEG-TK conjugation, mPEG-NH_2_ (100 mg), thioketal intermediate (127 mg), EDC (139 mg), and NHS (172.5 mg) were dissolved in dimethylformamide (DMF, 10 mL). The reaction was carried out under a nitrogen atmosphere using microwave synthesis at 80 °C for 3 h to allow efficient amide bond formation between the carboxyl groups of TK and the amine group of mPEG. The reaction mixture was subsequently purified by dialysis using a membrane (MWCO 1000 Da) against water/methanol (80:20, v/v), followed by deionized water to remove residual solvent and unreacted reagents. The purified PEG-thioketal intermediate (PT) was obtained with a yield of approximately 65–80%.

#### 2.2.3 Synthesis of PEG-TK-C18 (PTC polymer)

The PEG-TK intermediate (100 mg) was further reacted with stearyl amine (C18, 80 mg) using EDC/NHS coupling chemistry. Specifically, PEG-TK (100 mg), C18 amine (80 mg), EDC (240 mg), and NHS (145 mg) were dissolved in DMF (10 mL), followed by the addition of triethylamine (TEA, 80 μL) to facilitate the reaction. The mixture was stirred at 80 °C for 150 min under a nitrogen atmosphere. The reaction mixture was subsequently purified by dialysis using a membrane (MWCO 12–14,000 Da) against water, followed by deionized water to remove residual solvent and unreacted reagents. The purified product was lyophilized to obtain PEG-TK-C18 (PTC) as a solid. The overall yield of the final PTC polymer was approximately 50–70%. PTC structure was confirmed via ^1^H nuclear magnetic resonance spectroscopy (^1^H NMR, 400 MHz; Bruker, USA) in deuterated chloroform (CDCl_3_). Morphology and elemental composition of atorvastatin-loaded nanocarriers were examined using field emission transmission electron microscopy (FE-TEM; JEM-2100F, JEOL, USA).

#### 2.2.4 ROS scavenging activity of PTC-statin

The ROS scavenging capability of the nanoparticles toward superoxide anion radicals (O_2_•^-^), hydrogen peroxide (H_2_O_2_), and hydroxyl radicals (•OH) was comprehensively characterized using complementary fluorescence and colorimetric assays, as described below:

##### 2.2.4.1 Hydrogen peroxide (H_2_O_2_) scavenging assay

The H_2_O_2_ scavenging activity of the nanoparticles was evaluated using a tris(4,7-diphenyl-1,10-phenanthroline)ruthenium(II) dichloride (Ru(dpp)_3_) fluorescence-based assay. Briefly, different concentrations of PTC and PTC-Statin nanoparticles (0-500 μg/mL) were prepared in PBS. The nanoparticle samples were incubated with 1 mM H_2_O_2_-containing reaction solution followed by addition of the Ru(dpp)_3_ fluorescent probe under dark conditions at room temperature.

##### 2.2.4.2 Hydroxyl radical (•OH) scavenging assay

The hydroxyl radical (•OH) scavenging activity was assessed using a 3,3′,5,5′-tetramethylbenzidine (TMB) oxidation assay under Fenton-like conditions. In this system, hydroxyl radicals generated from the Fenton reaction (FeSO_4_) oxidize TMB to produce a blue-colored oxidized product measurable at 652 nm.

##### 2.2.4.3 Superoxide (O_2_•^-^) scavenging assay

The superoxide scavenging activity of the nanoparticles was evaluated using a nicotinamide adenine dinucleotide (NADH)–phenazine methosulfate (PMS)–water-soluble tetrazolium-1 (WST-1) assay. Briefly, different concentrations of PTC and PTC-Statin nanoparticles (0-500 μg/mL) were prepared in PBS. The reaction mixture consisted of NADH solution, PMS solution, WST-1 reagent, and nanoparticle samples. After incubation at room temperature for 10 min under dark conditions, the absorbance was measured at 450 nm using a microplate reader.

The superoxide scavenging activity was calculated using:

$$Scavenging\;Activity\;\left(\%\right)=\frac{A\_control-A\_sample}{A\_control}X\;100$$

where $A_{control}$ represents the absorbance of the radical-generating system without nanoparticles and $A_{sample}$ represents the absorbance in the presence of nanoparticles.

### 2.3 Drug loading, stability and release studies

PTC with atorvastatin calcium or IR780 (a near-infrared fluorescent dye) was dissolved in anhydrous dimethyl sulfoxide (DMSO) and added dropwise to distilled water (DW) under probe sonication (7 min, 30% amplitude). The suspension was dialyzed in deionized water for 12 h to remove residual solvent. Atorvastatin loading was quantified using high-performance liquid chromatography (HPLC). The colloidal stability of the nanoparticles was assessed by monitoring hydrodynamic diameter via dynamic light scattering (DLS) in PBS, serum, and DW over 15 days. Drug release was evaluated under physiological (phosphate-buffered saline, pH 7.4) and oxidative stress conditions (PBS containing 1 mM H_2_O_2_). Statin-loaded PTC dispersions were placed in dialysis bags (MWCO 3.5 kDa) and incubated at 37 °C with gentle shaking (100 rpm). Samples collected over 72 h were analyzed using HPLC.

### 2.4 *In vitro* experiments

Murine BV-2 microglial cells were obtained from ATCC (USA). High-glucose Dulbecco's Modified Eagle's Medium (DMEM), fetal bovine serum (FBS), and antibiotics (penicillin-streptomycin) were purchased from commercial suppliers. Assay kits included the MTS cell viability kit (Promega, USA), DCF-DA ROS detection kit (Abcam, UK), lipid peroxidation (MDA) assay kit (Elabscience, USA), ELISA kits for TNF-α and IL-6 (Invitrogen, USA), ABclonal ELISA kit for IL-10 (ABclonal, USA), and colorimetric assay kit for total superoxide dismutase (T-SOD; Elabscience Biotechnology, China). Measurements were performed using a fluorescence microscope (Evos FL Auto 2, Life Technologies, USA) and a microplate reader (Tecan, Switzerland).

#### 2.4.1 Cell culture

BV-2 microglial cells were cultured in high-glucose DMEM supplemented with 10% FBS and 5% antibiotics at 37 °C in a 5% CO_2_ incubator. Primary microglia were isolated from the cerebral cortices of neonatal Sprague-Dawley rats. The cortical tissues were mechanically dissociated and enzymatically digested with 0.25% trypsin for 15 min at 37 °C. The resulting cell suspension was filtered and centrifuged to collect the glial cells. The cells were then cultured in DMEM/F12 medium supplemented with 10% FBS and 1% penicillin-streptomycin in T75 flasks.

#### 2.4.2 Cell viability

Cells were seeded in 96-well plates (1 × 10^4^ cells/well), treated with PTC formulations for 24 h, and viability was assessed using the MTS assay following the manufacturer's instructions. Absorbance was measured at 492 nm.

#### 2.4.3 Oxygen-glucose deprivation (OGD)

Ischemia was simulated by incubating cells in glucose-free DMEM under hypoxia (1% O_2_, 5% CO_2_) for 4 h, followed by 24 h reperfusion in complete medium.

#### 2.4.4 Cellular uptake

Cells treated with IR780-loaded PTC (with or without OGD) were fixed, stained with 4′,6-diamidino-2-phenylindole (DAPI), and examined using fluorescence microscopy.

#### 2.4.5 ROS and lipid peroxidation

Intracellular ROS were measured by DCF-DA staining, and MDA levels were determined as an indicator of lipid peroxidation.

#### 2.4.6 Cytokine analysis

Supernatants were collected and analyzed for TNF-α, IL-6, and IL-10 using ELISA kits, following the manufacturer's protocols.

#### 2.4.7 BBB penetration assay

Human cerebral microvascular endothelial cells (hCMEC/D3) were cultured in Endothelial Cell Basal Medium-2 (EBM-2; Lonza, Switzerland) in rat collagen-coated plates. Cells were seeded onto polyethylene terephthalate (PET) membrane inserts (0.4 μm) in Transwell plates, and transendothelial electrical resistance (TEER) values were measured regularly. IR780-loaded PTC nanoparticles were then added to the upper chamber. Samples from the lower chamber were collected, and fluorescence intensity was measured at 780 nm using a fluorescence microplate reader.

#### 2.4.8 Hemolysis assay

Heparinized mouse blood was washed with PBS and red blood cells (RBCs) were collected. The RBCs were diluted and incubated with PTC-Statin, PBS, or DW for 30 min. After incubation, the suspension was centrifuged at 3000 × g for 5 min, and the absorbance of the supernatant was measured at 540 nm.

### 2.5 *In vivo* experiments

Male 8-week-old Sprague-Dawley rats (body weight 253–288 g) were obtained from Samtaco Bio (South Korea). Isoflurane was used for anesthesia. T2-weighted magnetic resonance imaging (MRI) scans were performed using a M7™ Compact MRI system (Aspect Imaging, Israel), and infarct volumes were quantified with Medical Image Processing, Analysis, and Visualization (MIPAV) software (NIH, USA). RNA isolation kits (TRIzol™, Invitrogen, USA), antibodies for Western blotting, and 5-bromo-2-deoxyuridine (BrdU) for immunofluorescence were purchased from commercial suppliers.

#### 2.5.1 Animal model and treatment

Transient middle cerebral artery occlusion (MCAO) was induced by filament insertion for 90 min, followed by reperfusion for 90 min under isoflurane anesthesia, as previously described [17]. Body temperature was maintained at 36.6 ± 0.5 °C during the procedure. Rats with neurological deficit scores (NDS) of 2-3 were selected and randomly assigned to groups receiving saline, atorvastatin calcium (10 mg/kg), blank PTC, or atorvastatin-loaded PTC (equivalent to 10 mg/kg atorvastatin). Treatments were administered daily via tail-vein injection for 7 days. All procedures were approved by the Institutional Animal Care and Use Committee of Chonnam National University (CNUHIACUC-22033).

#### 2.5.2 Brain MRI

T2-weighted MRI scans were performed on Days 1, 3, 5, and 7 following MCAO surgery. Infarct volumes were quantified using MIPAV software. Day 1 MRI data served as the individual baseline reference for each animal's initial infarct volume. Statistical analysis evaluated the relative change in infarct volume at Days 3, 5, and 7 as a ratio to the Day 1 baseline, enabling direct assessment of temporal changes across the treatment period.

#### 2.5.3 Behavioral evaluation

Motor coordination and balance were assessed using a rotarod test. Animals were placed on a rotating rod, and the latency to fall was recorded (five trials, maximum 300 s). Three 5-min pre-training sessions were conducted prior to the main test [21]. Motor performance was assessed at two post-operative time points, specifically at baseline (Day 1) and after treatment (Day 7) [22]. To support the validity of our behavioral results, rotarod tests were analyzed in parallel with MRI-confirmed infarct volume measurements obtained from the same animals, allowing a paired within-animal assessment of structural and functional outcomes. Neurological performance was evaluated using NDS. Animals were evaluated for motor, sensory, reflex, and balance abnormalities according to a standardized scoring system.

Sensorimotor asymmetry was evaluated using the corner test as previously described [23]. Two vertical boards (30 × 20 × 1 cm) were placed at a 30° angle to form a corner. Each rat was allowed to enter the corner, where both sides of the vibrissae were simultaneously stimulated by the boards. The rat then reared and turned either to the left or right to exit the corner. A total of 10 trials were performed for each rat with an interval of approximately 30 s between trials. The number of turns made toward the ipsilateral side of the brain lesion was recorded. Results were expressed as the percentage of ipsilateral turns relative to the total number of turns, which reflects sensorimotor asymmetry following stroke.

Sensorimotor function was assessed using the adhesive removal test [24]. Small adhesive tapes (approximately 3 × 3 mm) were placed on the palmar surface of each forepaw of the rat. After placement, the animal was immediately returned to its cage and observed. The latency to detect the adhesive (first attempt to touch or shake the paw) and the latency to remove the adhesive were recorded. Each rat was tested in three trials, and the mean latency for each measure was calculated across trials. A maximum cutoff time of 120 s was applied if the animal failed to remove the tape. Differences between the contralateral (affected) and ipsilateral (unaffected) forepaws were used to evaluate sensorimotor deficits following stroke.

#### 2.5.4 Molecular and histological analyses

On Day 7, total RNA was extracted from ischemic hemispheres for quantitative reverse-transcription polymerase chain reaction (qRT-PCR) analysis of inflammatory and neuronal markers using glyceraldehyde-3-phosphate dehydrogenase (GAPDH) as the internal reference. Western blotting was performed for proteins associated with tight junction integrity, vascular permeability, and inflammatory signaling. To specifically evaluate the anti-inflammatory phenotype switch, the expression levels of CD206 were determined in primary microglial cultures through incubation with primary antibodies against CD206. Vascular leakage was evaluated using Evans blue dye followed by fluorescence imaging. Neurogenesis and apoptosis were assessed by BrdU incorporation and immunostaining for neuronal nuclei (NeuN) and doublecortin (DCX), following standard protocols.

For immunofluorescence staining, brain sections were incubated overnight at 4 °C with the following primary antibodies: mouse anti-cleaved caspase-3 (CC3, 1:100, Thermo Fisher Scientific, catalog no. MA3-100) and mouse anti-CCAAT/enhancer-binding protein homologous protein (CHOP, 1:100, Cell Signaling Technology) for apoptosis assessment, rabbit anti-platelet-derived growth factor receptor beta (PDGFR-β, 1:100, Thermo Fisher Scientific) as a pericyte marker, and rabbit anti-glial fibrillary acidic protein (GFAP, 1:200, Thermo Fisher Scientific) as an astrocyte marker. Following three washes with PBS (5 min each), sections were incubated with species-matched fluorophore-conjugated secondary antibodies for 1 h at room temperature. Hematoxylin and eosin (H&E) staining was performed and imaged using a ZEISS Axio Scan.Z1 slide scanner to evaluate histopathological changes in the ischemic brain tissue.

#### 2.5.5 Evans Blue assay

BBB permeability was assessed using the Evans Blue extravasation assay. Rats were intravenously administered a 4% Evans Blue solution at a dose of 4 mL/kg body weight. Following a 2-h circulation period, animals were anesthetized with isoflurane and ketamine-xylazine and transcardially perfused through the left ventricle with 20 mL of cold phosphate-buffered saline (PBS) to clear residual intravascular dye. Brains were then harvested and dissected into ipsilateral and contralateral hemispheres. Each tissue sample was weighed and homogenized in 400 μL of 50% trichloroacetic acid/formamide to extract the dye. Following centrifugation at 10,000 × g for 20 min at 4 °C, the supernatant was collected and absorbance was measured at 620 nm using a spectrophotometer/fluorescence was measured using a spectrofluorometer with excitation at 620 nm and emission at 680 nm. The amount of extravasated Evans Blue was calculated based on a standard curve and expressed as μg of dye per gram of brain tissue.

#### 2.5.6 Bio-distribution

MCAO rats (n = 4) received IR780-loaded PTC nanocarriers via intravenous injection. At 6, 12, 24, 48, and 96 h post-administration, major organs including the brain, liver, spleen, and kidneys were isolated, imaged, and quantified using the FOBI imaging system (NEO Science, Suwon, South Korea).

#### 2.5.7 Intravenous administration and blood sampling for drug kinetics study

To evaluate the *in vivo* pharmacokinetics of the statin formulations, mice (average body weight of 25 g) were anesthetized with isoflurane and ketamine-xylazine. For the PTC-statin and statin groups, a dose of 10 mg/kg was administered intravenously via the retro-orbital venous sinus in a total volume of 100 μL. This route was selected to ensure rapid and consistent systemic entry of the nanocarrier and free drug formulations. For pharmacokinetic profiling, blood samples were collected at designated time points (0.5, 1, 12, 24, and 48 h). Retro-orbital blood sampling was performed by gently displacing the globe of the eye and inserting a capillary tube into the medial canthus to access the venous sinus. Following collection, the samples were analyzed using HPLC to determine the atorvastatin concentration (expressed in ng/mL).

### 2.6 Statistical analysis

All data were analyzed using GraphPad Prism 9 software. Normality of distribution was assessed with the Shapiro-Wilk test, and variance homogeneity using Levene's test. Parametric data were compared using analysis of variance (ANOVA) with appropriate post hoc tests, whereas non-parametric datasets were analyzed using the Kruskal-Wallis test. Repeated-measures data (e.g., NDS, rotarod performance) were analyzed using two-way ANOVA with sphericity corrections applied. A *p*-value < 0.05 was considered statistically significant.

## 3 Results

### 3.1 Characterization of ROS-responsive PTC nanocarriers

**Fig. 2:**
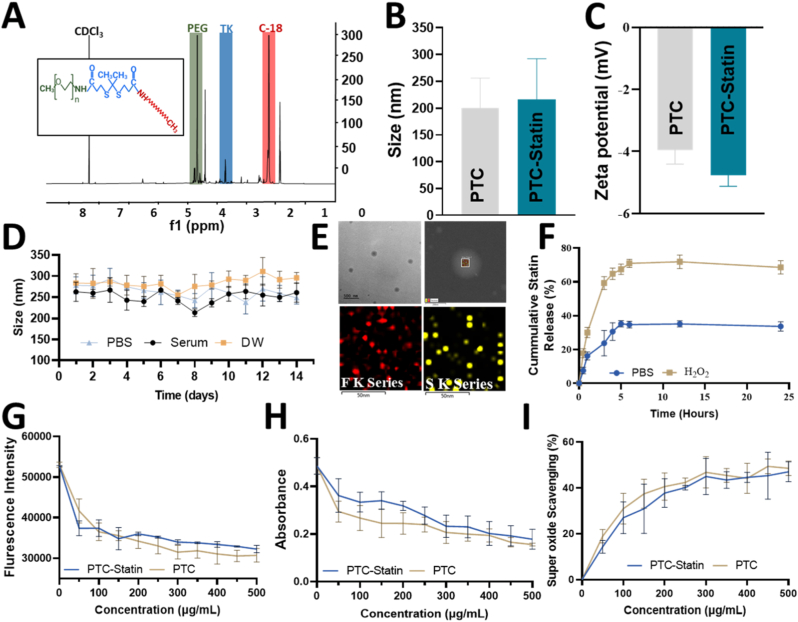
**Nanoparticle characterization.** (A) NMR spectrum confirming the chemical structure of PTC. (B) Size distribution and (C) zeta potential of PTC and statin-loaded PTC. (D) Stability of PTC under different conditions. (E) TEM image of statin-loaded PTC nanoparticles showing micellar structure and particle size, with EDAX analysis confirming atorvastatin loading into PTC. (F) Atorvastatin calcium release in PBS with and without ROS stimuli (1 mM H_2_O_2_, pH 7) at 37 °C. (G) H_2_O_2_ Scavenging Assay. (H) Hydroxyl radical scavenging assay (TMB oxidation). (I) Superoxide scavenging assay. NMR, nuclear magnetic resonance; TEM, transmission electron microscopy; PBS, phosphate-buffered saline; TK, thioketal; PEG, polyethylene glycol; PTC, polyethylene glycol-thioketal-C18.

The NMR spectrum of PTC confirmed its chemical structure by identifying key molecular components (Fig. 2A). A peak at 1.6 ppm corresponded to CH_3_-CH_3_ groups of the thioketal linker, while peaks at 2.6 and 2.8 ppm corresponded to CH_2_-CH_2_ groups from the same linker. Peaks at 3.4 and 3.6 ppm confirmed CH_3_ and CH_2_-CH_2_ groups from mPEG amine, and a peak at 1.2 ppm confirmed the C18 chain, together demonstrating its successful conjugation with PTC. The average hydrodynamic diameter of PTC nanoparticles was 210 ± 15 nm, whereas statin-loaded PTC particles were slightly larger at 230 ± 25 nm (Fig. 2B), with polydispersity indices of 0.231 and 0.245, respectively. The zeta potential of the final nanoparticles was −5 mV (Fig. 2C), indicative of colloidal suspension stability. Drug loading efficiency was 15 ± 3%, confirming successful incorporation of atorvastatin into the nanocarrier. As shown in Fig. 2D, the nanoparticles maintained stable size distributions across all tested conditions. In serum, the hydrodynamic diameter remained within 230-270 nm, with no significant increase over time, indicating resistance to protein-induced aggregation. Similarly, in PBS, the hydrodynamic diameter remained stable, demonstrating robustness under physiologically relevant ionic strength. TEM images further verified the micellar structure and particle size of statin-loaded PTC, and EDAX analysis confirmed atorvastatin loading (Fig. 2E).

As shown in Fig. 2F, the release profile of atorvastatin calcium exhibited characteristic biphasic behavior under physiological conditions. In PBS (pH 7.4, 37 °C), an initial rapid release phase of approximately 40% was observed within the first 5 h, followed by a sustained plateau with no further significant increase. This plateau is attributable to the stable retention of the remaining drug fraction within the hydrophobic C18 core, where strong hydrophobic interactions establish a thermodynamic equilibrium that limits passive diffusion in the absence of ROS. In contrast, the presence of H_2_O_2_ (1 mM) induced significantly accelerated and more complete drug release, confirming the ROS-responsive behavior of PTC-statin (Fig. 2F).

H_2_O_2_ scavenging activity was assessed using the Ru(dpp)_3_ fluorescence probe. As shown in Fig. 2G, the fluorescence intensity decreased progressively with increasing concentrations of PTC-statin (0-500 μg/mL), indicating effective quenching of H_2_O_2_. Specifically, the fluorescence intensity decreased from approximately 52,000 arbitrary units (AU) in the control group to approximately 32,000 AU at 500 μg/mL, demonstrating clear concentration-dependent scavenging. Similarly, the absorbance of oxidized TMB at 652 nm decreased with increasing nanoparticle concentrations, confirming effective inhibition of •OH-mediated oxidation (Fig. 2H). The absorbance decreased from approximately 0.48 in the control group to approximately 0.18 at 500 μg/mL for PTC-statin.

Superoxide scavenging activity was evaluated using the NADH-PMS-WST-1 assay. As shown in Fig. 2I, both PTC and PTC-statin exhibited concentration-dependent superoxide scavenging activity, reflected by a progressive decrease in WST-1 absorbance with increasing nanoparticle concentration. Scavenging efficiency reached approximately 49% and 47% inhibition for PTC-statin and PTC, respectively, at 500 μg/mL. Both formulations showed comparable superoxide scavenging efficiency, with no statistically significant difference between PTC and PTC-statin.

### *3.2* *In vitro* studies of ROS-responsive PTC statin

**Fig. 3:**
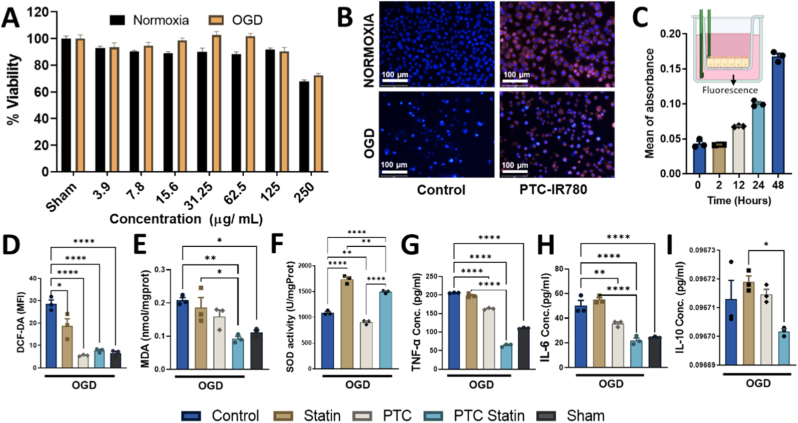
***In vitro* evaluation of PTC-statin in BV-2 cells.** (A) Cell viability under normoxic and OGD conditions, assessed via the MTS assay. (B) Nanoparticle uptake after 4 h incubation with IR780-loaded PTC, visualized via fluorescence imaging (scale bar = 100 μm). (C) BBB penetration assay (D) ROS suppression within OGD-treated cells, detected with DCF-DA staining. (E) Lipid peroxidation quantified through MDA levels in cell lysates. (F) SOD specific activity quantified in cell lysates. (G-I) Anti-inflammatory responses measured as TNF-α, IL-6, and IL-10 levels in culture supernatants of treated and untreated groups. Data are presented as mean ± SEM (n = 3), with significance determined using one-way ANOVA (∗*p* < 0.05; ∗∗*p* < 0.01; ∗∗∗*p* < 0.001; ∗∗∗∗*p* < 0.0001). OGD, Oxygen–glucose deprivation; PTC, polyethylene glycol-thioketal-C18; MDA, malondialdehyde.

Nanoparticles with different statin concentrations were evaluated for cytocompatibility in BV-2 cells using the MTS assay after 24 h (Fig. 3A). Cell viability remained above 80% at the highest concentration (125 μg/mL); therefore, 100 μg/mL was selected as the working dose for subsequent experiments. Fluorescence imaging confirmed efficient nanoparticle uptake under both normoxic and OGD conditions (Fig. 3B). BBB penetration of IR780-loaded PTC nanoparticles was assessed using the hCMEC/D3 transwell model. Fluorescence intensity in the lower chamber increased progressively over the observation period, consistent with time-dependent translocation of nanoparticles across the endothelial monolayer (Fig. 3C). Oxidative stress was assessed by measuring ROS (Fig. 3D) and MDA levels (Fig. 3E) after treatment with PBS, free statin, PTC, PTC-statin, or sham. OGD alone markedly elevated ROS (28.59 units) and MDA (0.2081 nmol/mg protein) levels. In contrast, treatment with PTC-statin significantly reduced ROS (7.7 units) and MDA (0.0923 nmol/mg protein) levels, reaching values comparable to those observed in the sham group. Notably, ROS and MDA were reduced by 3.7-fold and 2.2-fold relative to the OGD group, respectively (*p* < 0.0001, *p* = 0.0054). PTC-statin treatment significantly restored SOD activity compared with PTC treatment alone, indicating enhanced antioxidant defense (Fig. 3F). These results confirm that the thioketal-linked PTC nanocarrier is ROS-responsive, selectively releasing atorvastatin in the oxidative environment of OGD-treated cells. Cleavable thioketal bonds react with intracellular H_2_O_2_, triggering drug release while simultaneously reducing ROS levels through carrier degradation. This dual mechanism significantly contributes to the observed antioxidant effects.

Compared to the OGD control, treatment with PTC-statin reduced TNF-α by approximately 3.2-fold and IL-6 by 2.2-fold (Fig. 3G–H). Free statin administration produced only minor effects compared with OGD, highlighting the superior efficacy of PTC-statin. Overall, PTC-statin exerts strong anti-inflammatory activity *in vitro*, significantly suppressing TNF-α and IL-6 (*p* < 0.0001). IL-10 concentrations showed only minimal variation among the groups, suggesting the experimental treatments exerted a more pronounced effect on oxidative stress regulation than on IL-10-mediated inflammatory responses (Fig. 3I).

These results highlight thioketal-linked PTC micelles as a dual-action strategy against oxidative stress and inflammation, thereby offering a promising nanoplatform for managing stroke-associated neuroinflammation.

### *3.3* *In vivo* study

#### *3.3.1* *In vivo* MR imaging

**Fig. 4:**
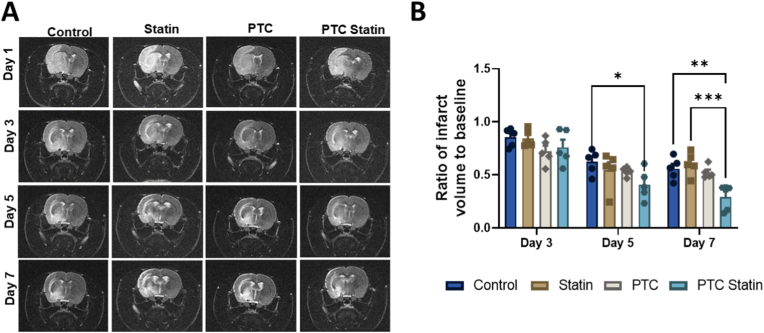
***In vivo* MRI assessment of ischemic infarct volume.** (A) Representative T2-weighted MR images of rat brains obtained every other day from day 1 to 7. (B) Quantitative analysis of infarct volume in ischemic regions on days 3, 5, and 7, showing a significant reduction in the PTC-statin group beginning at day 5. Day 1 served as baseline. Data are presented as mean ± SEM (n = 5). Statistical significance was determined using two-way ANOVA (∗*p* < 0.05; ∗∗*p* < 0.01; ∗∗∗*p* < 0.001; ∗∗∗∗*p* < 0.0001). PTC, polyethylene glycol-thioketal-C18.

Infarct volume was evaluated from T2-weighted MR scans obtained every other day from day 1-7. Treatment groups exhibited markedly reduced infarct volumes compared with controls (Fig. 4A). At day 7, the infarct ratio was significantly lower in the PTC-statin group (*p* = 0.0032) than in the control, whereas no significant improvement was observed in the PTC or free-statin groups (Fig. 4B). Longitudinal analysis showed that only the PTC-statin group achieved a significant reduction by day 5 (*p* = 0.0249) compared with controls. By day 7, the infarct ratio in the PTC-statin group was also substantially lower than that in the free-statin group (*p* = 0.0004).

#### 3.3.2 Behavioral study

**Fig. 5:**
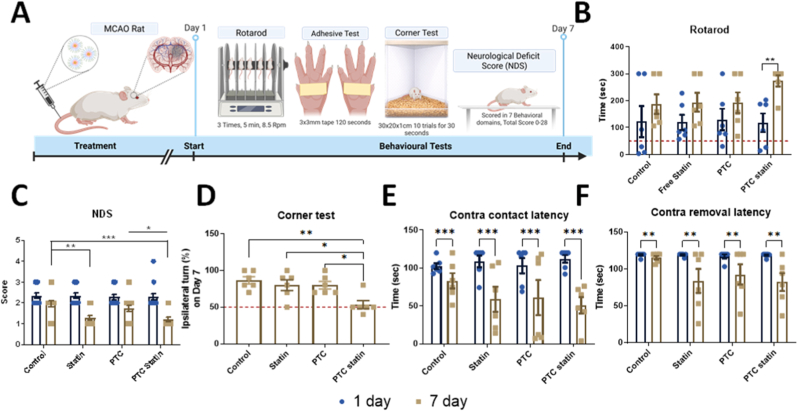
**Behavioral and motor performance evaluation.** (A) Schematic of the behavioral tests performed on MCAO rats on days 1 and 7. (B) Rotarod testing demonstrated improved coordination, balance, and endurance in PTC-statin-treated rats, with a significant difference compared with controls (*p* = 0.002). Values are presented as ranges (minimum to maximum); n = 6. (C) NDS for each group on days 1 and 7 are expressed as mean ± SEM (n = 15). Sphericity was evaluated using Mauchly's test, and when this assumption was not met, violations were corrected using the Greenhouse-Geisser method. (D) The corner test demonstrated the sensorimotor asymmetry in the treated groups, n = 6. (E and F) Adhesive removal test results showing sensorimotor deficits, with a statistically significant difference observed in contact latency, n = 6. Statistical significance was set at *p* < 0.05 (∗*p* < 0.01; ∗∗*p* < 0.001; ∗∗∗*p* < 0.0001).

Behavioral and motor performance was evaluated using the rotarod test, NDS, corner test, and adhesive removal test (Fig. 5A). To address the relationship between structural infarct size and functional motor performance, rotarod latency was assessed at Day 1 and Day 7 post-MCAO and analyzed in parallel with MRI-confirmed infarct volume from the same animals. PTC-statin-treated rats performed significantly better than the control group on Day 7 (*p* = 0.002; Fig. 5B). In contrast, animals in the control group showed no significant changes on Day 1 or Day 7 in either rotarod or NDS analyses (Fig. 5C), as determined by Tukey's multiple comparison test. These findings demonstrate the therapeutic potential of the ROS-responsive thioketal-linked PTC platform in the context of post-ischemic motor recovery.

Corner test data (Fig. 5D) were used to assess sensorimotor asymmetry following stroke. The percentage of ipsilateral turns was nearly 100% in all groups on Day 1. On Day 7, the percentage of ipsilateral turns showed significantly greater improvement in the PTC-statin group compared with the control, statin, and PTC groups. In contrast, there was no significant difference between the control group and either the statin group or PTC group, nor was a significant difference observed between the PTC group and the statin group.

Sensorimotor deficits were further evaluated using the adhesive removal test (Fig. 5E–F). The detection latency, defined as the time from adhesive placement until the animal's first contact with the tape, was significantly reduced between Day 1 and Day 7 across treatment groups, reflecting improvement in somatosensory function. Similarly, the removal latency, defined as the time from adhesive placement until complete tape removal, decreased in all treatment groups from Day 1 to Day 7, indicating progressive recovery of fine motor function. The reduction in removal latency was most prominent in the PTC-statin group, suggesting that PTC-statin treatment promoted functional recovery.

#### 3.3.3 qRT-PCR for pro-inflammatory markers

**Fig. 6:**
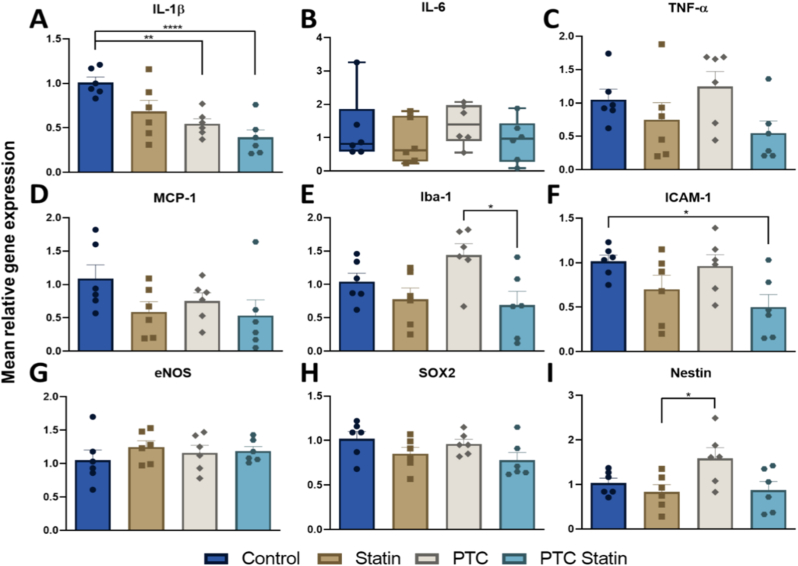
**RT-PCR analysis of inflammatory mediators, adhesion molecules, and neural progenitor markers.** mRNA levels of (A) IL-1β, (B) IL-6, (C) TNF-α, (D) MCP-1, (E) ICAM-1, (F) SOX2, (G) Iba-1, (H) eNOS, and (I) Nestin were quantified. Data are presented as ranges (minimum to maximum, n = 6). Statistical significance was determined using one-way ANOVA or the Kruskal-Wallis test, with thresholds of *p* < 0.05 and ∗*p* < 0.01. PTC, polyethylene glycol-thioketal-C18.

The mRNA expression levels of pro-inflammatory cytokines, adhesion molecules, and neural markers were assessed by qRT-PCR following PTC-statin treatment (Fig. 6). IL-1β expression was significantly decreased in the PTC-statin group compared with the control group (*p* < 0.01, Fig. 6A). IL-6 expression did not differ significantly across groups (Fig. 6B). TNF-α and MCP-1 expression were similarly reduced in the PTC-statin group compared with the control group (Fig. 6C and D), consistent with suppression of the pro-inflammatory response. Iba-1, a marker of microglial activation, was markedly reduced in the PTC-statin group compared with the PTC group (*p* < 0.05; Fig. 6E). ICAM-1, a cell adhesion molecule reflecting endothelial inflammation, was downregulated in the PTC-statin group compared with the control group (*p* < 0.05; Fig. 6F). eNOS expression was modestly elevated across all treatment groups relative to the control, although no statistically significant differences were observed (Fig. 6G). SOX2 expression was similar across all groups (Fig. 6H). Nestin, a marker of neural progenitor cells, was markedly elevated in the PTC group relative to all other groups, whereas the PTC-statin group showed expression levels comparable to those of the control group (Fig. 6I).

#### 3.3.4 Western blotting

**Fig. 7:**
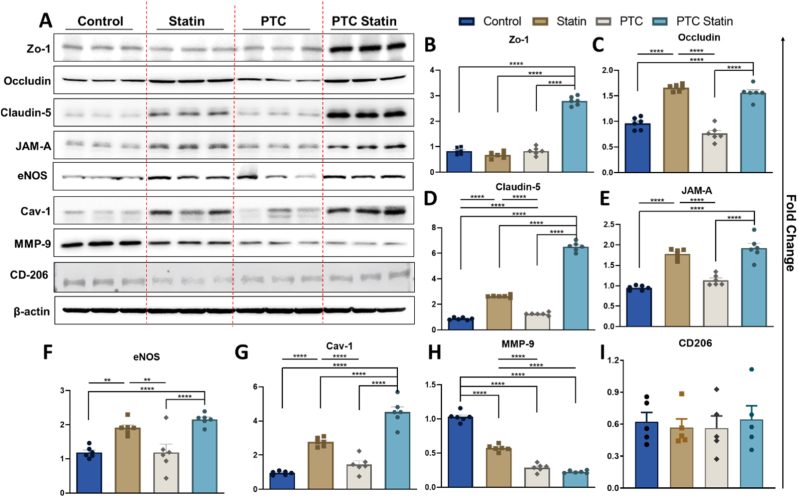
**Western blot for tight junction proteins, endothelial markers, and microglial polarization.** (A) Representative bands for ZO-1, occludin, claudin-5, JAM-A, eNOS, cav-1, MMP-9, and CD206. (B-E) Quantification of tight junction proteins. (F-G) Quantification for endothelial markers. (H-I) Quantification of MMP-9 proteolytic activity and M2 microglial polarization marker. Data are presented as mean ± SEM (n = 6, except n = 5 for CD206) and were analyzed using one-way ANOVA. Significance thresholds: *p* < 0.05; ∗*p* < 0.01; ∗∗*p* < 0.001; ∗∗∗*p* < 0.0001. PTC, polyethylene glycol-thioketal-C18.

PTC-statin treatment markedly elevated tight junction protein expression, consistent with restoration of BBB integrity (Fig. 7A). ZO-1, a cytoplasmic scaffolding protein, was significantly upregulated in the PTC-statin group compared with all other groups (Fig. 7B). Occludin expression was highest in both the statin and PTC-statin groups, whereas the PTC-only group showed the lowest expression among all conditions (Fig. 7C), suggesting that carrier-mediated drug delivery, rather than PTC alone, is required for transmembrane tight junction recovery. Claudin-5 and JAM-A, which are essential for tight junction strand assembly and endothelial sealing, respectively, were also most prominently elevated in the PTC-statin group (Fig. 7D and E).

Regarding endothelial markers, eNOS expression significantly increased in the PTC-statin group compared with the control and PTC groups, with the highest levels observed in the PTC-statin group (Fig. 7F), indicative of enhanced nitric oxide bioavailability and improved cerebrovascular tone. Cav-1 expression followed a similar pattern, with the PTC-statin group exhibiting significantly higher expression than all other groups (Fig. 7G), consistent with caveolae-mediated stabilization of endothelial barrier function. These findings were paralleled by a progressive reduction in MMP-9 levels across treatment groups, with the PTC-statin group demonstrating the lowest expression (Fig. 7H), indicating attenuation of extracellular matrix proteolysis and tight junction degradation. Although CD206 expression in primary microglia showed a modest numerical trend toward elevation in the PTC-statin group, this difference did not reach statistical significance (Fig. 7I).

#### *3.3.5* *In vivo* imaging system for BBB integrity

**Fig. 8:**
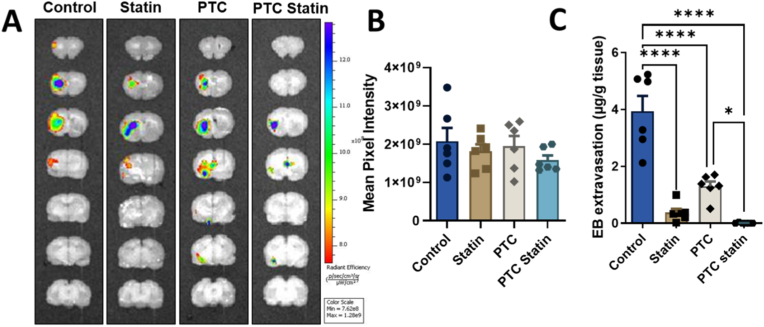
**IVIS® imaging for BBB integrity.** (A) Representative IVIS® fluorescence images of rat brains on Day 7, showing the spatial distribution of BBB disruption in the ischemic hemisphere. (B) Quantification of fluorescence accumulation in ischemic rat brain regions using IVIS®. (C) Quantification of Evans blue extravasation as an index of BBB permeability. Data are presented as mean ± SEM (n = 6) and were analyzed using one-way ANOVA followed by Tukey's post hoc test. Statistical thresholds: ∗*p* < 0.05; ∗∗*p* < 0.01; ∗∗∗*p* < 0.001; ∗∗∗∗*p* < 0.0001. PTC, polyethylene glycol-thioketal-C18; BBB, blood-brain barrier; IVIS, *in vivo* imaging system.

*In vivo* imaging system (IVIS®) assessment revealed the highest fluorescence radiance in the control group, followed by the PTC, free-statin, and PTC-statin groups (Fig. 8A). Mean fluorescence radiance in the control group (2 × 10^9^ p/s/cm^2^) was numerically greater than that in the free-statin (1.8 × 10^9^ p/s/cm^2^), PTC (1.9 × 10^9^ p/s/cm^2^), and PTC-statin (1.5 × 10^9^ p/s/cm^2^) groups. However, no statistically significant differences were detected across groups (Fig. 8B). The adjusted p values were as follows: control vs. free-statin, *p* = 0.7967; control vs. PTC, *p* = 0.9701; and control vs. PTC-statin, *p* = 0.3693.

In contrast, Evans blue extravasation provided a more sensitive index of BBB permeability, revealing a significant reduction in dye accumulation in the ipsilateral hemisphere of the PTC-statin group (0.007 μg/g) compared with the free-statin (0.378 μg/g), PTC (1.293 μg/g), and control (3.938 μg/g) groups (Fig. 8C), indicating marked attenuation of BBB hyperpermeability and restoration of vascular integrity following ischemic stroke.

#### 3.3.6 Immunofluorescence imaging for apoptosis and neurogenesis

**Fig. 9:**
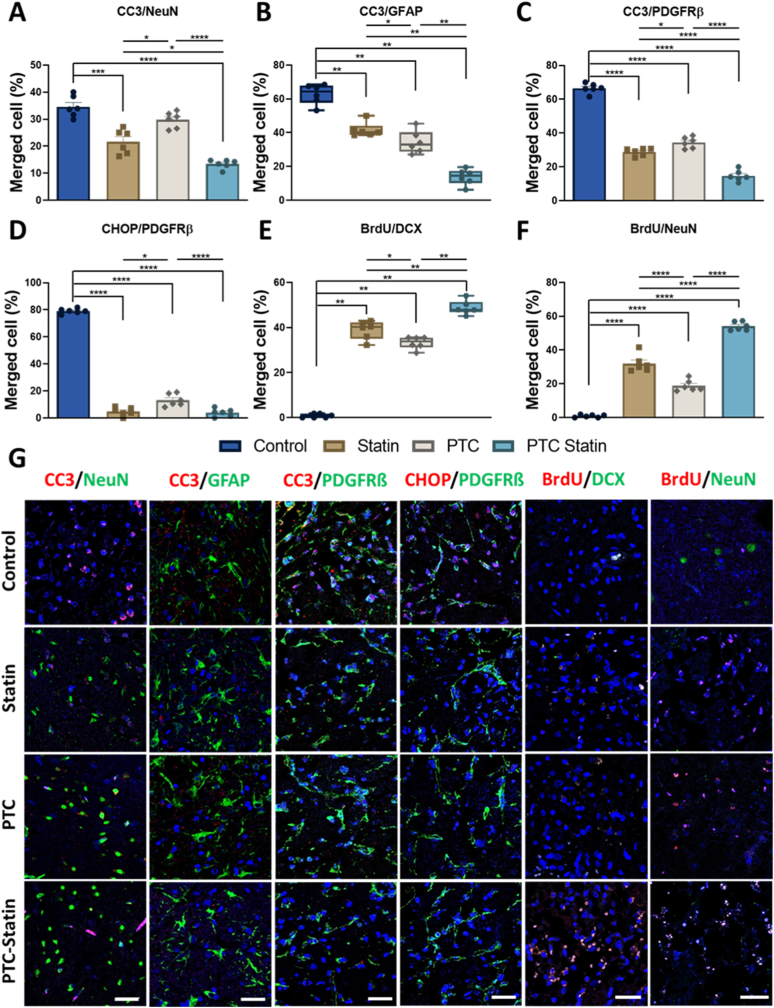
**Immunofluorescence analysis of neuronal apoptosis and neurogenesis.** (A) Quantification of apoptotic neurons in the ischemic frontal cortex penumbra using CC3/NeuN co-staining, showing a significant reduction on day 7 (p < 0.0001). (B) Astrocyte survival assessed via CC3/GFAP staining. (C) Pericyte apoptosis assessed via CC3/PDGFRβ staining. (D) Evaluation of pericyte preservation against ER stress-induced dysfunction examined via CHOP/PDGFRβ co-staining. (E) Neurogenesis measured via BrdU/DCX staining at day 14, showing enhanced neuronal generation. (F) BrdU/NeuN staining at day 28 confirmed increased neuronal survival. (G) Representative immunofluorescent images from ischemic penumbral sections (Scale bar = 20 μm). Statistical analysis was performed with one-way ANOVA (n = 6); ∗*p* < 0.05, ∗∗*p* < 0.01, ∗∗∗*p* < 0.001, ∗∗∗∗*p* < 0.0001. ER, endoplasmic reticulum; PTC, polyethylene glycol-thioketal-C18.

The immunofluorescence analysis revealed that PTC-statin treatment produced marked neuroprotective effects and effectively mitigated the secondary injury cascade as illustrated in Fig. 9. We performed double staining for CC3 and NeuN to identify apoptotic neurons and the quantitative results showed the lowest percentage in the PTC-statin group at 13.42% while the control group reached 34.60%. The free-statin group also exhibited a reduced apoptotic ratio of 21.66% although this effect was less pronounced than the potent neuroprotection observed in the PTC-statin group. Furthermore, staining for BrdU and DCX demonstrated robust neurogenesis, with newly generated neurons at day 14 accounting for 48.75% in the PTC-statin group. In contrast, the control group showed only 0.75% whereas the free-statin and PTC groups recorded 38.93% and 33.25% respectively.

Astrocytic activation, which is a key component of the inflammatory response following ischemia, was confirmed through immunolabeling for GFAP. The subsequent analysis of CC3 and GFAP co-staining showed that PTC-statin significantly enhanced astrocyte survival after the ischemic insult. Additionally, the pericyte marker PDGFRβ highlighted the role of these cells in maintaining vascular stability and supporting the neurovascular recovery mentioned in our therapeutic strategy. The evaluation of the CC3 and PDGFRβ combination confirmed that ischemic conditions compromise vascular stability by inducing pericyte apoptosis. We also evaluated CHOP as a marker of endoplasmic reticulum stress-induced apoptosis to determine whether such stress drives pericyte dysfunction. The experimental data demonstrated that PTC-statin treatment effectively mitigated this dysfunction and preserved a higher population of healthy pericytes compared with the other groups. These results underline the ROS-responsive function of the thioketal-linked PTC system where thioketal bonds selectively degrade in the presence of H_2_O_2_. This mechanism triggers targeted micelle destabilization and drug release in ischemic regions to provide the dual benefits of site-specific atorvastatin delivery and direct ROS neutralization.

The assessment of long-term neuronal survival on day 28 using BrdU and NeuN staining revealed a marked increase in newly generated neurons in the PTC-statin group at 54.10% while the control group remained at 0.9067%. Although the difference was less pronounced when compared with the free-statin group at 31.98%, PTC-statin still achieved superior outcomes. Collectively, the evidence of increased neurogenesis and improved neuronal survival in the PTC-statin group highlights the therapeutic potential of this ROS-responsive delivery strategy for addressing persistent oxidative damage and promoting recovery in ischemic stroke.

#### 3.3.7 Biodistribution, drug kinetics and Toxicity Studies

**Fig. 10:**
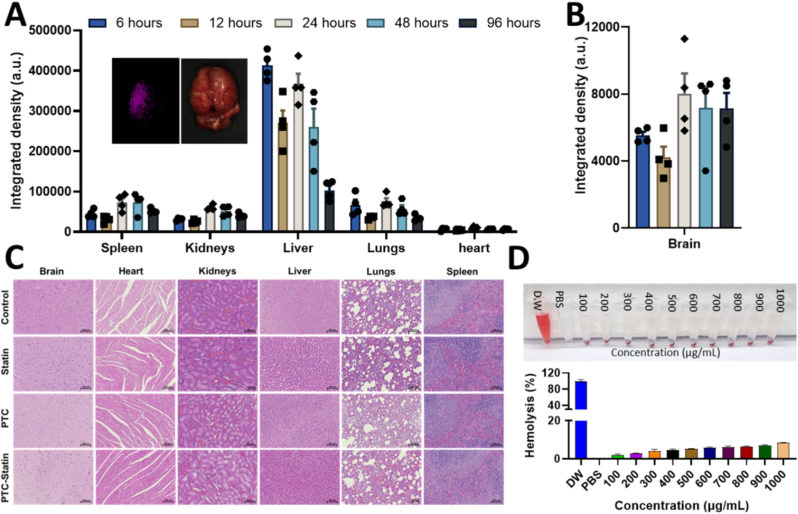
Bio-distribution, Drug kinetics and Toxicity Studies. (A) Quantification of fluorescence accumulation in primary organs. Inset image of Brain-where fluorescence accumulation is seen in the ROS rich region(Ischemic). (B) Quantification of fluorescence accumulation in Brain (C) H&E staining of Primary organs (D) Hemolysis activity of PTC-statin.

The *Ex vivo* imaging analysis demonstrated the time-dependent biodistribution of the PTC nanocarriers across major organs (Fig. 10A). At the 6-h time point, strong fluorescence was observed in the liver along with lower fluorescence in the spleen and kidneys. This pattern indicates predominant uptake by the reticuloendothelial system while maintaining limited off-target distribution due to the protective PEG corona. Notably, detectable fluorescence remained in the brain up to 96 h after administration with preferential accumulation in the ROS-rich ischemic region compared with the contralateral hemisphere (Fig. 10A–B). This region-specific accumulation is likely attributable to BBB disruption and increased vascular permeability following ischemic stroke working in tandem with the ROS-responsive release mechanism of the PTC nanocarrier system.

The pharmacokinetic results demonstrated that the nanocarrier effectively modulated *in vivo* drug release and systemic availability by enhancing systemic exposure and reducing systemic clearance. The PTC-statin formulation exhibited a 9.38-fold increase in AUC at 19,982.4 ± 31,089.0 ng h/mL compared with the free drug at 2131.4 ± 825.3 ng h/mL. This finding indicates significantly improved drug retention in the systemic circulation. The observed clearance for the PTC-statin group was approximately 3-fold lower than the clearance of the free drug at 0.0019 versus 0.0057 mL/h/kg. These data confirm that the PEGylated nanocarrier successfully circumvents rapid elimination. The apparent volume of distribution also decreased to 0.34 times that of the free drug which indicates enhanced plasma retention that increases the opportunity for targeted accumulation over time (Table 1).

**Table 1:** Pharmacokinetic parameters of free drug (Statin) and nanocarrier formulation (PTC-Statin) after IV administration in mice (n = 6).

Parameter	Unit	Free Drug (Statin)	Nanocarrier (PTC-Statin)	Fold Change (PTC-Statin/Statin)
AUC_0−t_	ng·h/mL	2131.4 ± 825.3	19,982.4 ± 31,089.0	9.38x
CL	mL/h/kg	0.0057 ± 0.0028	0.0019 ± 0.0017	0.33x
Vz	(mg/kg)/(ng/mL)	0.0702 ± 0.0487	0.0237 ± 0.0306	0.34x

Histological analysis of major organs using H&E staining showed no significant morphological abnormalities in the brain, heart, liver, spleen, lung, or kidney in the treated animals compared with controls, indicating excellent systemic biocompatibility of the treatment (Fig. 10C). The hemolysis assay was performed using red blood cells incubated with varying concentrations of PTC-statin. The results showed negligible hemolysis across all tested concentrations which further supports the safety profile of the nanomicelle formulation for intravenous administration (Fig. 10D).

## 4 Discussion

This study demonstrates that atorvastatin calcium-loaded PTC nanocarriers respond to ROS in the infarct region to trigger localized drug release and a cascade of protective effects. In *in vivo* experiments, PTC-statin nanoparticles were administered once daily for 7 days via the tail vein in MCAO rats, with MRI performed to monitor infarct volume reduction. Treatment improved motor function recovery and decreased inflammatory cytokine levels and neuronal apoptosis within the ischemic penumbra. The formulation also enhanced endothelial function and preserved BBB integrity. Furthermore, apoptosis was reduced in neurons and astrocytes as well as pericytes while neurogenesis increased in the subventricular zone. These results support the potential of ROS-responsive PTC nanocarriers for neuroprotection in ischemic stroke and warrant further investigation toward clinical translation.

Nanoparticle-based formulations improve stability and bioavailability along with BBB penetration which enables antioxidant agents to overcome the limitations of conventional therapies [25,26]. ROS-responsive nanoparticles offer a promising approach to reducing oxidative injury and improving clinical outcomes following ischemic stroke [27,28]. Preclinical studies in animal models of cerebral ischemia have demonstrated efficacy in limiting oxidative stress and neuronal apoptosis as well as brain edema and infarct size [26,29,30]. Polymers such as PLGA, PEG, and PLA are widely employed to enhance brain targeting and delivery efficiency [31]. As highlighted in recent studies, ROS-responsive systems improve therapeutic precision by minimizing off-target effects and enhancing drug accumulation at diseased sites [32,33]. Additionally, PEGylation confers steric stabilization and stealth properties [34,35], extending systemic circulation time [17,36]. While PEG length alters transcytosis and brain targeting efficiency [37], the linker length kinetics also affect nanoparticle uptake and penetration [38].

The PTC-statin platform leverages the oxidative microenvironment of ischemic tissue to achieve site-specific activation whereas conventional statin-loaded nanocarriers such as liposomes rely primarily on passive delivery. Although thioketal-based ROS-responsive systems have been previously reported, most studies focus exclusively on ROS-triggered drug release. The present platform integrates a dual-functional mechanism by combining ROS-responsive drug release with intrinsic ROS-scavenging through thioketal bond cleavage, thereby addressing both targeted delivery and oxidative stress simultaneously. Critically, the thioketal core ensures that drug release is spatially restricted to the ROS-rich ischemic infarct, reducing off-target exposure and maximizing therapeutic efficiency at the lesion site [32,33]. The biphasic release profile observed under physiological conditions provides further evidence of this structural integrity. The diffusion-limited plateau at approximately 40% cumulative release reflects intact thioketal crosslinks that preserve micellar architecture in the absence of oxidative stimuli, thereby minimizing premature drug leakage during systemic circulation. Upon exposure to pathological ROS levels, thioketal bond cleavage induces progressive micellar destabilization, enabling on-demand drug mobilization at the target site. In a prior study, PTC was shown to alleviate renal inflammation and apoptosis in murine ischemia-reperfusion models by protecting against ROS-induced injury in human proximal tubular epithelial cells, establishing the platform's cytoprotective utility beyond the CNS context [16].

The physicochemical properties of PTC micelles provide a mechanistic basis for their *in vivo* applicability. The PEGylated nanosystem with a size of approximately 250 nm demonstrated improved biocompatibility and prolonged systemic circulation [16]. The PEG corona confers stealth properties by minimizing opsonization and shielding the nanoparticles from immune recognition. This effect extends their residence time in systemic circulation and increasing the probability of accumulation at the ischemic lesion. Minor fluctuations in size observed across all media were attributed to dynamic interactions with proteins or ions rather than irreversible aggregation. No progressive increase in particle size was detected under any condition, indicating that the nanoparticles maintain structural integrity and dispersion stability over prolonged periods. These findings confirm that the nanoplatform exhibits excellent colloidal stability in biologically relevant environments, which is essential for reliable *in vivo* performance. Although the zeta potential of the PTC micelles was approximately −5 mV, below the conventional threshold for electrostatically stabilized dispersions, this profile is consistent with PEGylated nanocarriers in which steric, rather than electrostatic, forces govern colloidal stability. The dense hydrophilic PEG corona generates a protective hydration layer around each micelle, providing steric repulsion and minimizing interparticle interactions to prevent aggregation under physiological conditions. PEG chain length further modulates transcytosis efficiency and brain-targeting capacity [35]. The relatively narrow polydispersity index (PDI) values (0.23–0.24) support a uniform particle size distribution, reinforcing the overall colloidal stability of the formulation. Collectively, nanoparticles within this size range have been previously demonstrated to facilitate passive accumulation at sites of disrupted BBB integrity via the EPR-like effect in cerebrovascular injury. The stealth properties conferred by PEGylation further amplify this mechanism by prolonging the window of lesion exposure [39]. Consistent with this mechanistic rationale, *in vitro* BBB penetration assessed using the hCMEC/D3 transwell model demonstrated time-dependent translocation of IR780-loaded PTC nanoparticles across the endothelial monolayer, providing direct experimental evidence of the nanoparticles' capacity to traverse the BBB under physiologically relevant conditions.

The ROS responsiveness of PTC enables effective drug release in the H_2_O_2_ environment. Atorvastatin was selected for its ability to reduce infarct volume and mitigate inflammation while supporting BBB and endothelial function recovery [[40], [41], [42], [43], [44]]. The ROS-scavenging behavior observed in Fig. 2G and H is consistent with the chemical characteristics of thioketal linkers which are selectively cleaved by strong oxidative species such as H_2_O_2_ and •OH. This cleavage reaction not only consumes ROS but also contributes to the ROS-responsive degradation of the nanostructure. The concentration-dependent decreases in both fluorescence and absorbance signals (Fig. 2G and H) confirm that PTC-statin functions as an active ROS-scavenging system. Furthermore, the plateau observed at higher concentrations (400–500 μg/mL) suggests near-saturation of ROS quenching under the experimental conditions. Notably, both PTC and PTC-statin exhibited comparable superoxide scavenging efficiency across the tested concentration range, indicating that the ROS-scavenging capability primarily originates from the TK-containing polymeric framework. This observation highlights the intrinsic antioxidant nature of the linker which contributes significantly to the overall performance of the nanoplatform.

While thioketal-based ROS-responsive systems have been previously reported [[14], [15], [16]], most studies primarily focus on ROS-triggered drug release. In contrast, the PTC-statin platform integrates a dual-functional mechanism, combining ROS-responsive drug release with intrinsic ROS-scavenging capability through thioketal degradation. Furthermore, compared to conventional statin-loaded nanocarriers such as liposomes, which rely mainly on passive delivery, our system leverages the oxidative microenvironment of ischemic tissue to achieve site-specific activation. In previous studies, liposomes and bilirubin-based ROS-responsive micelles loaded with atorvastatin calcium [17] show significant infarct volume reduction, improved neurological function, and enhanced anti-inflammatory effects. In this study, the H_2_O_2_-responsiveness of PTC is evident from the significant infarct reduction, decreased oxidative stress markers, and enhanced BBB repair. Degradation of thioketal linkers likely contributes to neuroprotection by directly neutralizing H_2_O_2_. PTC-statin nanoparticles (∼230 nm) significantly reduce ROS, lipid peroxidation, and anti-inflammatory cytokines in *in vitro* OGD models compared with statin alone and control. While all treatments lowered ROS compared to controls, only PTC-statin produced significant reductions in lipid peroxidation, TNF-α, and IL-6, highlighting the benefit of combinatorial therapy. PTC-statin nanoparticles reduced infarct volumes approximately 2-fold by day 5 and 3-fold by day 7 compared with the control and statin groups. The poor bioavailability of the drug may explain its poor performance when compared with the nanosystem [45].

Ischemia-induced BBB disruption increases permeability, allowing immune cell infiltration that contributes to tissue damage and secondary injury. Western blot analysis shows that PTC-statin treatment significantly improved BBB integrity compared to the control, statin, and PTC groups. Increased ZO-1 indicates restored BBB integrity, while higher occludin levels reflect BBB repair. Elevated Claudin-5 and JAM-A levels indicate tight junction restoration and reduced inflammation, respectively. Increased eNOS promoted nitric oxide production, vasodilation, and cerebral blood flow, indicating restored perfusion, while reduced MMP-9 indicated decreased BBB disruption and inflammation. IVIS® imaging confirmed improved BBB integrity in the PTC-statin group, consistent with enhanced coordination and endurance observed in behavioral tests on days 1 and 7.

During ischemic stroke, reperfusion elevates ROS levels, triggering neuronal apoptosis [46]. Immunofluorescence staining for CC3, NeuN, GFAP, PDGFR-β, CHOP, BrdU, and DCX was performed to evaluate neuroprotection, BBB structural restoration, and neurogenesis in ischemic brain regions. On Day 7, the expression ratios of CC3/NeuN, CC3/GFAP, CC3/PDGFR-β, and CHOP/PDGFR-β were significantly lower in the statin, PTC, and PTC-statin groups compared with the Control group, supporting treatment efficacy against ischemia-induced cell death. Critically, PDGFR-β and GFAP staining provided direct histological evidence of BBB structural repair. This was evidenced by significantly greater perivascular PDGFR-β+ pericyte coverage and restored GFAP + astrocyte end-feet association in PTC-statin-treated animals. These observations were consistent with the markedly reduced Evans Blue extravasation. BrdU/DCX and BrdU/NeuN analyses further demonstrated enhanced neurogenesis, with all treatment groups showing significant increases relative to the Control group at Day 14 and Day 28. Collectively, PTC-statin provided the greatest benefit, combining ROS suppression, reduced apoptosis, restored BBB integrity, and neurogenesis stimulation, underscoring the therapeutic potential of ROS-responsive thioketal nanocarriers in ischemic stroke. Histopathological staining revealed no abnormality in major organs following seven doses of administration, and hemolysis percentages remained well below the commonly accepted safe threshold of 5%, confirming excellent blood compatibility and indicating that the nanoparticles do not induce membrane disruption or erythrocyte damage.

Building on the neuroprotective effects described above, PTC-statin treatment significantly improved motor function, as demonstrated by rotarod performance. In the acute phase, animals exhibit severe neurological deficits driven not only by irreversible infarct core damage but also by perilesional edema, glutamate-mediated excitotoxic spreading, and diaschisis, defined as a transient functional depression of regions remote from but structurally connected to the infarct [47]. As perilesional edema resolves and diaschisis reverses over the course of days 3 through 7, meaningful functional recovery can occur even in animals with structurally large infarcts. Therefore, the partial motor recovery at Day 7 is an expected finding that has been reported in previous studies [21,22,48,49]. Rotarod latency at Day 7 was significantly improved only in the PTC-statin group, demonstrating a treatment-specific enhancement of functional recovery that extends beyond the spontaneous neurological improvement observed in the control group. These findings collectively support the translational potential of PTC-statin as a promising, mechanistically targeted therapeutic approach capable of enhancing functional motor recovery in patients with ischemic stroke.

This study has limitations. First, although BV-2 microglial cells were utilized to investigate microglial polarization, they do not fully recapitulate the complexity of primary microglia or the *in vivo* brain microenvironment. Nonetheless, additional Western blot analysis of CD206 expression in primary microglial cultures showed a trend toward upregulation in the PTC-statin treated group, providing molecular evidence of an M2-phenotype shift in primary cells. This finding, along with the MCAO model data, supported the validity of the *in vitro* conclusions. The validation of primary microglia remains a priority objective for future studies to further investigate the temporal dynamics of microglial polarization. Second, while the present study demonstrates that PTC-statin modulates oxidative stress and inflammatory responses, the biomarker panel used here is relatively limited, focusing only on total ROS and MDA as oxidative stress indicators and TNF-α and IL-6 as inflammatory markers. Although these are widely accepted and inflammmatory indicators, they do not fully represent the complexity of redox balance and inflammatory signaling networks. The observed restoration of SOD activity alongside minimal changes in IL-10 suggests that the treatment primarily enhances enzymatic antioxidant capacity rather than inducing a broad anti-inflammatory response. Future studies should incorporate a broader panel of oxidative stress and inflammatory biomarkers to more comprehensively elucidate the pharmacological actions of PTC-statin. Third, we did not assess the *in vivo* ROS-sensitivity and therapeutic efficiency depending on local ROS levels at the lesion site. In addition, although *ex vivo* fluorescence imaging using IR780-conjugated PTC nanocarriers demonstrated time-dependent biodistribution, whole-body *in vivo* optical imaging was not performed. Consequently, real-time spatiotemporal trafficking of the nanocarriers in the living animal could not be directly visualized. Future investigations will incorporate *in vivo* whole-body optical imaging alongside blood-pool sampling to establish rigorous pharmacokinetic parameters and further validate the targeted delivery capability of the PTC nanocarrier system. Fourth, interspecies anatomical, physiological, and genetic differences may limit the translation of these findings to humans. Furthermore, the absence of direct *in vivo* long-term fate tracking of the PEG-nanocarriers is a limitation of this study. Future work will include longitudinal *in vivo* imaging and quantitative tissue analyses to define long-term fate and safety for clinical application. PEGylation of ROS-responsive PTC-statin extended circulation time and facilitated BBB penetration. Consequently, this platform holds strong potential for targeted therapy in acute ischemic stroke and ROS-associated vascular disorders. Future studies should focus on optimizing novel nanoparticle designs to precisely enhance targeting and specificity thereby overcoming biological barriers in clinical settings.

## 5 Conclusion

This study successfully developed dual functional ROS-responsive PEGylated thioketal micelles encapsulating atorvastatin for the targeted treatment of ischemic stroke. Compared with conventional free statin therapy, the PTC-statin system exhibited significantly enhanced neuroprotective efficacy within the MCAO model. Our findings demonstrate that the formulation effectively reduces infarct volume and improves both neurological and motor recovery while attenuating inflammatory responses and restoring BBB integrity. The ROS-responsive thioketal platform enabled microenvironment triggered drug release while simultaneously scavenging excessive reactive oxygen species to provide a synergistic therapeutic strategy for ischemic injury. Furthermore, the stimulation of neurogenesis and neuronal survival observed in this study underlines the translational potential of this platform. Collectively, these findings suggest that advanced ROS-responsive nanomedicine platforms such as PTC-statin represent a promising approach for improving clinical outcomes in stroke therapy.

## Funding

This work was supported by the 10.13039/501100001321National Research Foundation (NRF) of Korea Grant funded by the Korean Government (grant number NRF-2023R1A2C2006414).

## CRediT authorship contribution statement

**Raveena Nagareddy:** Conceptualization, Data curation, Investigation, Visualization, Writing – original draft. **Aravindkumar Sundaram:** Investigation, Visualization, Writing – original draft. **Ja-hae Kim:** Conceptualization, Data curation, Investigation, Visualization. **Ji hye Kim:** Formal analysis, Investigation, Visualization. **Shyam Vasvani:** Data curation, Formal analysis, Validation. **Seho Kweon:** Data curation, Formal analysis, Investigation. **In-Kyu Park:** Conceptualization, Data curation, Investigation, Visualization, Writing – review & editing. **Yong Yeon Jeong:** Conceptualization, Data curation, Investigation, Visualization, Writing – review & editing. **Kang-Ho Choi:** Conceptualization, Data curation, Investigation, Visualization, Writing – review & editing.

## Declaration of competing interest

The authors declare the following financial interests/personal relationships which may be considered as potential competing interests: Kang Ho Choi reports financial support was provided by National Research Foundation (NRF) of Korea. If there are other authors, they declare that they have no known competing financial interests or personal relationships that could have appeared to influence the work reported in this paper.

## Data Availability

Data will be made available on request.

## Glossary

**Ischemic Stroke**: A condition caused by blocked blood flow to part of the brain, leading to tissue damage
**MCAO (Middle Cerebral Artery Occlusion)**: An experimental model of ischemic stroke created by blocking the middle cerebral artery in animals
**Nanocarriers**: Tiny engineered particles designed to transport drugs to specific sites in the body
**Neurogenesis**: The process of generating new neurons in the brain
**Oxidative Stress**: Cellular damage caused by excessive reactive oxygen species (ROS)
**PEGylation**: The process of attaching polyethylene glycol (PEG) chains to nanoparticles to improve stability, circulation, and biocompatibility
**Thioketal Linkers**: Chemical bonds that break down in the presence of ROS, enabling controlled drug release
